# Effects of manual therapies on stability in people with musculoskeletal pain: a systematic review

**DOI:** 10.1186/s12998-020-0300-9

**Published:** 2020-02-18

**Authors:** Julie C. Kendall, Dein Vindigni, Barbara I. Polus, Michael F. Azari, Samantha C. Harman

**Affiliations:** 1 Chiropractic, School of Health and Biomedical Sciences, RMIT University, Bundoora, VIC 3083 Australia; 2 School of Engineering, RMIT University, Bundoora, VIC 3083 Australia; 3 Private practice, Melbourne, Australia

**Keywords:** Ageing, Balance, Manual therapy, Pain, Systematic review

## Abstract

**Introduction:**

Chronic musculoskeletal pain is associated with reduced balance performance and falls risk. Manual therapies are commonly used interventions for musculoskeletal pain. There is emerging evidence that manual therapies may improve balance. The aim of this systematic review was to examine the effectiveness of manual therapies for musculoskeletal pain on measures of static and dynamic stability.

**Methods:**

Six electronic databases were searched using pre-defined eligibility criteria and two independent reviewers assessed all identified records. Risk of bias was assessed using the 12-item Cochrane Risk of Bias assessment by two authors independently and any discrepancies resolved through consensus. Meta-analysis was conducted when three or more studies used the same outcome measures including gait speed, timed up and go test, step test and sit-to-stand test.

**Results:**

Twenty-six studies were included in the analysis. Both spinal and extremity musculoskeletal pain conditions were represented. Manual therapies included manipulation, mobilisation and massage. The most common intervention compared to manual therapy was exercise. Outcome measures included both clinical and objective measures of stability. Overall the risk of bias was reported as generally low or unclear.

**Conclusion:**

Improvement in stability measures were reported in studies comparing manual therapy in the short term, but not long-term follow-up. There was no clear association between significant pain reduction and measures of stability. Further prospective studies are recommended to investigate whether manual therapies should be part of an integrative healthcare plan for risk of falls management and when a transition from manual therapy to more active interventions should occur for long term management.

## Introduction

The global population is ageing, as exemplified by recent Eurostat population data which estimates that the population of people aged 65 years and older will increase from 18% in 2013 to 28% in 2060 [1]. Ageing increases the risk of escalating morbidity (people living longer in poor health) [2].

Fall related injury in older people is a major health problem [3, 4]. Stiffer, less coordinated gait, poor balance control and decreased muscle strength have been cited as major causes of falls in older people [4, 5]. In addition, chronic musculoskeletal pain has been associated with previous history of a fall [6] and an increased occurrence of future falls [7]. Musculoskeletal pain in older adults is common and debilitating, with one in five older adults reporting that this pain interferes with normal life [8]. The Australian Institute of Health and Welfare reported that chronic back problems affect 27% of people aged between 65 and 74 years in Australia [9]. Furthermore, the severity and number of chronic musculoskeletal pain sites are associated with reduced balance performance and falls risk [10].

Manual therapy is a commonly used intervention for musculoskeletal pain, particularly low back pain (LBP) [11] and neck pain [12]. Two systematic reviews have found emerging evidence that manual therapy may improve balance [13, 14]. These systematic reviews included studies of asymptomatic and symptomatic participants with outcomes limited to falls and balance. They reported that improvements in balance were associated with reduction in pain intensity in symptomatic, but not in asymptomatic, participants. No meta-analyses were conducted in these systematic reviews, due to heterogeneity of the participants and outcome measures and the low methodological quality of the trials. An updated and expanded review of published studies in the literature needs to be conducted examining participants with pain.

Stability is a term that is used to denote how balance is controlled. If standing stability is perturbed, for example, various neurophysiological protective mechanisms need to be actioned in order to preserve whole body centre of mass within the base of support – that is, to preserve balance. Balance control deficits are associated with decreased stability. Clinical assessments of stability include tests of physical performance such as the sit-to-stand test or one-legged standing – which test balance control mechanisms. Balance impairment is a risk factor for falls as is gait impairment [5]. If pain reduction is associated with stability improvement, it is important that future trials assessing manual therapy for pain management are of high quality and include relevant functional physical performance measures [15] to assess these changes, even beyond static balance.

Therefore, the objective of this study was to examine the effectiveness of manual therapies for musculoskeletal pain on stability (including balance, physical performance and fear of falls). For the purposes of this review, reduced stability was measured and defined by: experiencing a fall; increased self-reported fear of falling; and reduced performance on objective measures of mobility and balance. This provides important information for researchers conducting future trials of manual therapies for musculoskeletal pain using clinical or objective static and dynamic stability outcome measures.

## Methods

### Types of studies

Included in this review were any controlled trials (randomised, quasi-randomised and non-randomised trials). Retrospective study designs, cohort studies, case reports, case series, commentaries, letters to the editor and expert opinions were excluded. Only English language studies were included.

### Types of participants

Participants in studies that met the inclusion criteria reported musculoskeletal pain of the spine or extremities. Diagnoses included, but were not limited to, neck pain, LBP, spinal pain, non-specific joint pain, fibromyalgia, arthritis, osteoarthritis (OA), disc herniation or any other bodily pain affecting the spine or extremities. Pain from multiple bodily sites was included. Diagnoses of musculoskeletal pain with radiating symptoms into the extremities, such as sciatica, were also included. Non-musculoskeletal pains such as that arising from referred visceral pain, malignancy, or nervous system pathology were excluded. Participants without pain, such as healthy participants were excluded. While stability and falls risk is predominantly of concern in older adults, in order to increase the reach of this review, any participants over the age of 18 were included.

### Types of interventions and comparisons

Studies using interventions were included if they comprised at least one component of manual therapy. Manual therapies involving manipulation (high velocity, low amplitude thrust techniques to improve joint movement), mobilisation (low velocity, low-to-high amplitude non-thrust techniques to improve joint movement), or massage (pressure and movement techniques to muscles and other soft tissues) were included. Trials of manual therapy in combination with other therapies, such as exercise, were also included. Comparison groups consisting of placebo, sham therapy, no treatment (wait-list control), and any other type of active intervention were included. All comparison interventions were pooled for meta-analysis, as there were not sufficient numbers of each individual intervention (eg. placebo) for comparison.

### Types of outcome measures

Outcome measures in studies that met the inclusion criteria consisted of number of falls, physical performance on clinical balance measures (such as sit-to-stand, gait speed, timed up and go (TUG) test), objective balance measures (including changes in centre of pressure on a force plate), and subjective measures of stability including psychological concerns of falling (such as fear of falls or falls efficacy questionnaires).

### Search methods

Electronic databases searched in Jan 2018 were The Cochrane Central Register of Controlled Trials (CENTRAL), Cochrane database of systematic reviews, PubMed, EMBASE, CINAHL, and Index to Chiropractic Literature. Keywords consisted of terms related to “manual therapy” combined with “falls” OR “balance” OR “physical performance” AND “musculoskeletal pain” (A full list of search terms is available from the corresponding author upon request). Search terms were modified for each database, and appropriate subheadings were used for each database searched.

### Study selection and data extraction

After duplicates were removed, two reviewers independently screened all titles and abstracts identified from the electronic database searches. Authors identified which studies should be further examined for inclusion into the review. Full text records were sourced, and two reviewers independently examined each record. Any disagreements between review authors were resolved by a third reviewer. Data extraction of included studies was carried out by at least two authors independently. Disagreements were resolved through consultation and involved a third reviewer if necessary.

### Risk of bias

Risk of bias was assessed using the 12-item Cochrane Risk of Bias assessment [16] by two authors independently and any discrepancies between authors were resolved through consensus.

### Data analysis

Studies selected for meta-analysis were those that comprised outcome measures utilised by more than two studies. Meta-analysis was performed in Review Manager 5.3. Short-term outcomes were those classified as immediate follow-up to 3 months. Long term outcomes were classified as greater than 3 months. Authors were contacted to access raw-data if there was insufficient detail in the published manuscript. Studies that were not able to be included in meta-analysis were included in the descriptive synthesis. Due to the high levels of heterogeneity between studies, a random effects model was used for all meta-analyses [17]. Heterogeneity in meta-analysis was measured with i^2^.

## Results

**Fig. 1:**
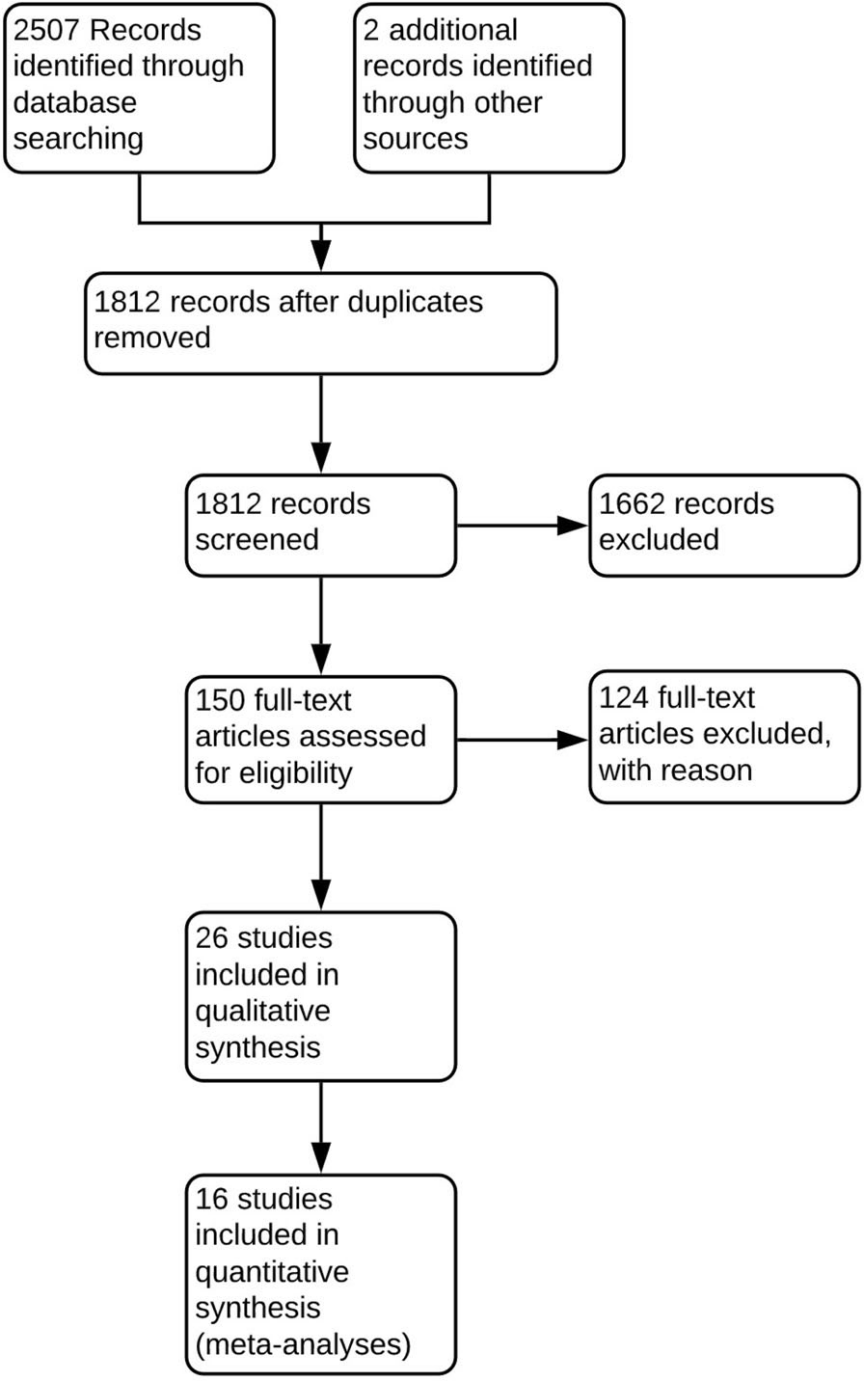
PRISMA flow chat of included studies. A total of 2509 records were screened, after excluding duplicates and studies that did not fulfil the inclusion criteria 25 studies were included for qualitative synthesis and 16 included in meta-analyses

Of 2509 citations reviewed, 150 were assessed for full text eligibility, 124 were excluded leaving 26 studies for inclusion [18–43] (Fig. 1). One hundred and twenty-four were excluded with reasons provided, which generally consisted of not including manual therapies as interventions, not including appropriate outcome measures, and study design. A list of reasons for exclusion can be given by contacting the authors.

Musculoskeletal pain diagnoses consisted of knee OA, hip OA, knee or hip OA, LBP, neck pain, knee pain, fibromyalgia, ankle arthropathy, and post-vertebral fracture (Table 1). Manual therapies consisted of manipulation [20, 30, 31, 36, 37, 39, 43], mobilisation [22, 28, 30, 31, 37, 39, 43] and massage [18–22, 26, 28, 29, 33, 37–40, 42] (Table 1).

**Table 1:** Description of included studies

Study	Pain region	Intervention group M(SD). Sham group 2 M(SD).	Intervention	Comparison	Pain Measure	Risk of falls measure
Bennell 2005 [21]	Hip OA	Physiotherapy group 67.4 (8.6). Placebo group 69.8 (7.5).	Physiotherapy (massage, taping, mobilisation) and self-management	Sham ultrasound	VAS	Step test
Bennell 2014 [20]	Hip OA	Intervention group 64.5 (8.6). Sham group 62.7 (6.4).	Mobilisation and/or soft tissue and/or manipulation plus exercise	Sham ultrasound	VAS	Sit-to-stand, step test, gait speed, 4-square step test
Beselga 2016 [23]	Hip OA	Intervention group 78.3 (6.3). Placebo group 77.5 (6.9)	Mobilisation of the hip	Simulated mobilisation	NRS11	TUG*, sit-to-stand*, gait speed*
Abbott 2013 [19]	Hip or Knee OA	Manual therapy + usual care group 67.3 (10.2). Exercise + usual care group 66.9(8.2). Usual care group 66.1 (10.7).	Mobilisation and/or soft tissue and/or manipulation and usual care	Exercise and usual care; usual care	NRS11*, WOMAC*	TUG, sit-to-stand, gait speed
Abbott 2015 [18]	Knee OA	Manual therapy + exercise group 61 (12). Exercise group 64 (10)	Mobilisation and/or soft tissue and/or manipulation plus exercise	Exercise	NRS11*, WOMAC*	TUG, sit-to-stand*, gait speed
Cheawthamai 2014 [25]	Knee OA	Manual therapy group 66.62 (8.77). Exercise group 64.05 (7.86).	Self-manual therapy and exercise	Exercise	VAS	Gait speed
Cortés Godoy 2014 [26]	Knee OA	Manual therapy group 85(median) (81–89(1st and 3rd quartiles)). Exercise group 84 (median) (82–84.5 (1st and 3rd quartiles)).	Massage and exercise	Exercise	VAS	TUG
Deyle 2000 [29]	Knee OA	Physiotherapy group 64 (9.9). Exercise group 62.2 (9.2)	Physiotherapy (mobilisation, massage) and exercise	Sub-therapeutic ultrasound	WOMAC	Gait speed
Deyle 2005 [28]	Knee OA	Physiotherapy group 64 (9.9). Exercise group 62.2 (9.2)	Physiotherapy (mobilisation, massage) and exercise	Exercise	WOMAC	Gait speed
Fitzgerald et al. 2016 [31]	Knee OA	Exercise + manual therapy group 58 (9.8). Exercise group 58.3 (10).	Manual therapy and exercise	Exercise	WOMAC(* at nine weeks) NRS11	TUG, sit-to-stand, gait speed
French 2013 [32]	Knee OA	Exercise + manual therapy group 58 (9.8). Exercise group 58.3 (10)	Manual therapy and exercise	Exercise	NRS11	Sit-to-stand, gait speed
Jardine 2012 [34]	Knee OA	Osteopathic group 63.20 (7.97). Control group 63.73 (9.63)	Osteopathic fascial release	No treatment	VAS	Step test
Lee 2017 [36]	Neck pain	Manual therapy + exercise group 59 (2.4). Exercise group 58 (1.6)	Therapeutic exercise with joint mobilisation applied to cervical and upper thoracic spine	Therapeutic exercise alone	VAS*, NDI*	Static balance ability
Maiers 2014 [37]	Neck pain	Manual therapy + exercise group 59 (2.4). Exercise group 58 (1.6).	Chiropractic (manipulation, mobilisation, traction, massage) and exercise	Exercise	NRS11*, NDI	TUG
Rudolfsson 2014 [38]	Neck pain	Massage group 51.2 (9.0). Neck coordination exercise group 50.7 (8.6). Strength training exercise group 51.6 (9.0).	Massage	Exercise	NRS11	Balance (COP)*
Bennell 2010 [22]	Spine pain	Physiotherapy group 66.2 (8.0). Control group 66.3 (11.8).	Mobilisation, massage, postural taping and exercise	No intervention	NRS11	TUG
Dougherty 2014 [30]	LBP	Manual therapy group 76.99 (6.77). Control group 77.04 (6.81)	HVLA spine manipulation and/or flexion distraction and/or mobilisation	Sham ultrasound	VAS, ODI*	TUG
Goertz et al., 2016 [43]	LBP	High velocity manipulation group 44.1 (10.6). Low velocity mobilisation group 44.5 (10.2). Control group 44.4 (10.5).	Spinal manipulation, spinal mobilisation	Sham control	None	Postural sway (blindfolded and on soft surface, without shoes); muscle activity (EMG) of paraspinal muscles in response to an unexpected sudden load.
Hicks et al. 2016 [33]	LBP	Intervention group 69.5 (7.0). Exercise group 70.7 (6.8)	Passive control intervention (heat, ultrasound and massage)	Trunk muscle training program (exercises) augmented with neuromuscular electrical stimulation	Modified Oswestry LBP Questionnaire NRS11	TUG test;* gait speed*; Tampa scale of kinesiophobia; global rating of functional improvement
Kim 2015 [35]	LBP	Manual therapy group 59.2 (6.5). Exercise group 62.6 (6.6)	Myofascial therapy and muscle energy technique plus exercise	Exercise	VAS*, ODI*	Balance system SD*
Ruhe 2012 [39]	LBP	Manual therapy group 39.8 (10.5). Control group 41.5 (5.5)	Manipulation, mobilisation and/or massage	No intervention	NRS11*	Balance (COP)*
Trampas 2014 [40]	LBP	Manual Therapy with massage and exercise 35.8 (7.16). Exercise group 33.4 (12.01)	Massage and exercise	Exercise	None	Biodex stability system
Yu et al. 2016 [42]	LBP	Myofascial release group 70.4 (3.2). Exercise group 69.4 (4.1)	Myofascial release of the iliopsoas	Exercises	NRS11	Balance (changes in pressure applied to each force plate and shows the stability of the centre of the gravity). Remodified Schober’s test (RST)
Castro-Sanchez 2011 [24]	Fibromyalgia	Myofascial therapy group 55.3. Sham ultrasound 53.5	Myofascial therapy	Sham ultrasound	McGill, VAS*, NRS11*	Postural stability
Cuesta-Barriuso 2014 [27]	Ankle arthropathy	Mobilisation of the ankle group 37.6 (13.1). Traction of ankles group 33.5 (11.7)	Mobilisation of the ankle, infrared thermotherapy, exercises, ice	Traction of ankles, infrared thermotherapy, exercises and ice	VAS	Romberg’s test
van den Dolder 2006 [41]	Knee pain	Massage group 55 (11). No intervention group 52 [18]	Massage	No intervention (wait list)	Patellofemoral pain severity questionnaire	Step test*

Table descriptions: *COP* centre of pressure, *GP* general practitioner*, LBP* low back pain, *NDI* neck disability index, *NRS11* numerical rating scale (scored 0–10), *OA* osteoarthritis, *ODI* Oswestry disability index, *TUG* timed up & go test, *VAS* visual analogue scale; WOMAC - Western Ontario and McMaster Universities arthritis index

*significant between group difference on this measure

Exercise was the most common intervention that was compared with manual therapy (Table 1) Exercise was prescribed as part of individual supervised programs [18, 21, 26, 27, 31–33, 36, 38, 40, 42], home exercise programs [25, 28, 29, 35], or a combination of supervised and home exercises [37]. Comparison interventions involved no treatment [22, 34, 39, 41], patients continuing with usual care [19], and sham interventions (detuned ultrasound [20, 21, 24, 30], sub-therapeutic ultrasound [29], or manipulative ‘set-up’ without thrust or mobilisation [23]).

Outcome measures consisted of clinical balance measures (gait speed, TUG, sit-to-stand, step test), balance performance (static balance, modified Schober’s test, force place centre of pressure, postural stability and Romberg’s) (Table 1). No studies that met the inclusion criteria were found that measured the number of falls, or psychological concerns of falling.

Studies selected for meta-analysis were those that comprised outcome measures utilised by more than two studies. These outcomes were gait speed, TUG test, step test and sit-to stand test.

Studies not included in meta-analysis were those examining balance [24, 27, 35, 36, 38–40, 43] (due to the heterogeneity of the balance measurements) and a further two studies because their published data were not in a format appropriate for meta-analysis (displayed in a graph [25] and median and interquartile ranges of non-normative data [26]). Authors were contacted for raw-data and did not respond. One study [32] was partially included in meta-analysis. It was included in the gait speed meta-analysis but could not be included in the sit-to-stand meta-analysis because this study measured the time taken to sit-to-stand five times, unlike the other studies which measured the number of stands in 30 s. While not included in meta-analyses, all these studies are included in the descriptive synthesis together with the other studies.

### Clinical balance measures

**Fig. 2:**
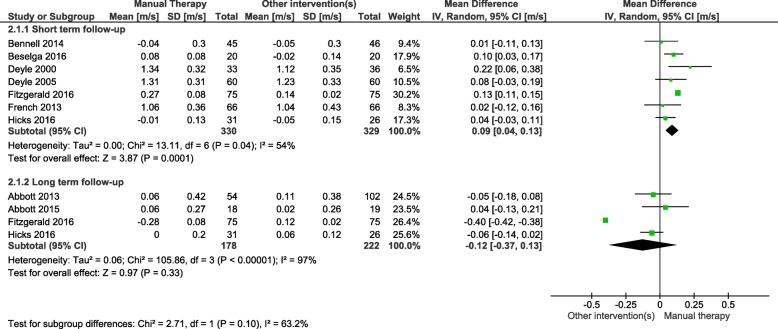
Gait speed meta-analysis: Interventions consisting of manual therapies were associated with significantly increased gait speed compared to other interventions in the short-term follow-up but not in longer term

**Fig. 3:**
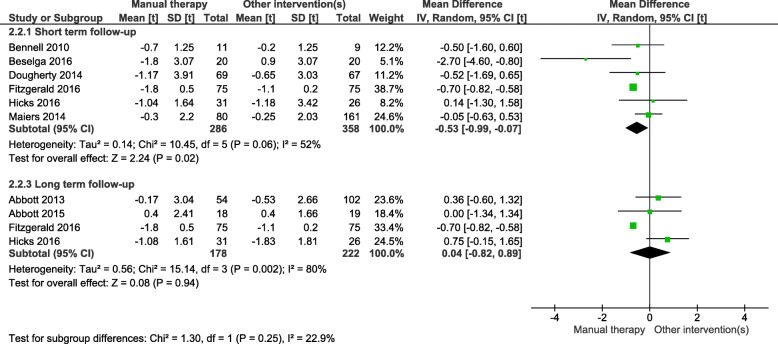
Timed Up and Go (TUG) meta-analysis: Interventions consisting of manual therapies were associated with significantly improved TUG test time compared to other interventions in the short-term follow-up but not in longer term

Manual therapy significantly improved gait speed and TUG test time compared to other interventions in short-term follow-up, however, not in in the long term (Figs. 2 and 3).

Gait speed improved by 0.09 m/s (95%CI 0.04, 0.13). However, there was one study that was not included in meta-analysis [25] that compared manual therapy to exercise, and found no significant difference in gait speed following manual therapies with home exercise versus home exercise alone in short-term follow-up.

Likewise, interventions including manual therapies found a significant improvement in TUG test scores of − 0.53 s (95%CI -0.99,-0.07), in the short but not long-term follow-up. However, there was one study that was not included in meta-analysis [26] that did not find any significant difference between combined manual therapy and exercise vs exercise alone on the get up and go test (a physical performance test similar to the TUG) in short-term follow-up.

There was a high level of heterogeneity in the studies (gait speed i^2^ = 54% *p* = 0.04, TUG i^2^ = 52% *p* = 0.06)). This heterogeneity was largely driven from the study by Fitzgerald and colleagues [31]. Removing this study from the meta-analyses, gait speed remained significant, while TUG became non-significant. Due to all studies having high levels of clinical heterogeneity, it was decided to leave the Fitzgerald and colleagues’ study in the meta-analysis. With such high levels of heterogeneity, all meta-analyses results should be interpreted with caution, and more research needs to be conducted to determine the short-term benefits of manual therapy compared to other interventions.

There was no significant difference between studies consisting of manual therapies compared to other interventions for the sit-to-stand test or step test.

### Objective balance measures

Eight studies examining balance measured objective balance [24, 27, 35, 36, 38–40, 42]. These studies were either too heterogeneous in the parameters analysed or there was insufficient reporting on the calculations used, to compare any of them in a meta-analysis.

Seven of these eight studies [24, 27, 35, 36, 39, 40, 42] used measures of pain perception and balance as an outcome of an intervention for musculoskeletal pain and recorded a reduction in pain perception as a result of the intervention. Note that Trampas and colleagues [40] used pain pressure threshold of muscular trigger points, not pain perception per se, as the outcome measure. Five studies noted an associated improvement in balance [35, 36, 39, 40, 42].

### Risk of bias

**Fig. 4:**
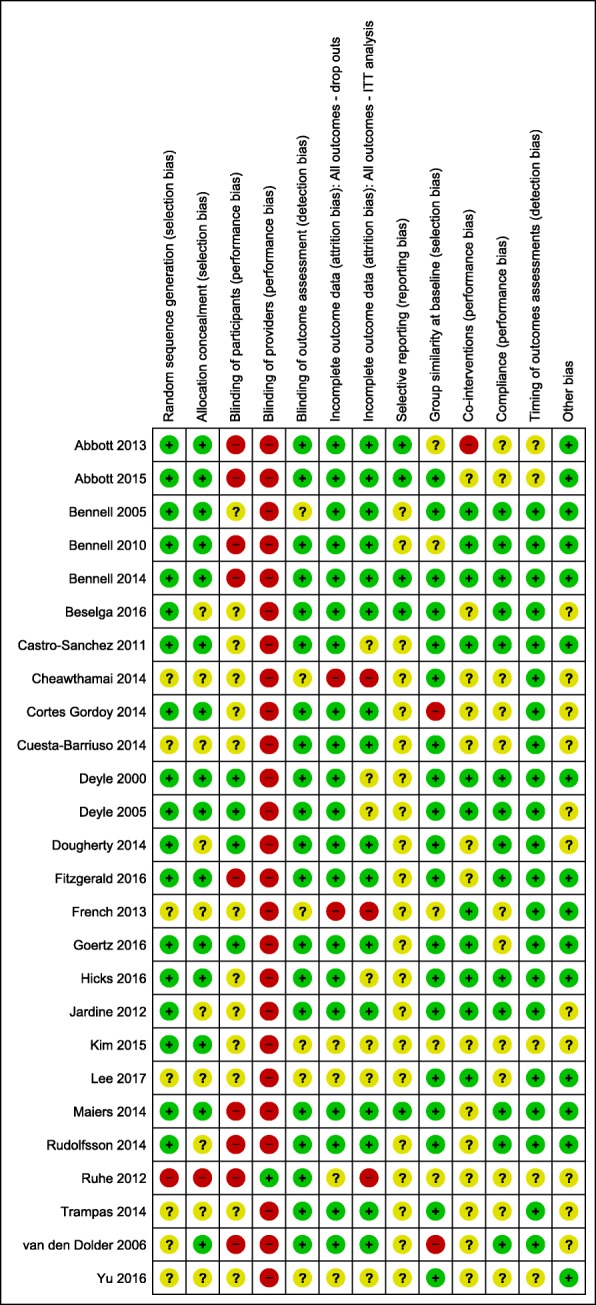
Risk of bias summary: review authors’ judgements about each risk of bias item for each included study

Except for performance bias, the overall analysis of risk of bias found the studies to be of a generally low or unclear level of bias (Fig. 4).

The risk of selection bias was low with: 18/26 papers using random sequence generation in their methodology; 15/26 papers reported using allocation concealment; and group similarity at baseline found in 19/26 papers. There was blinding of participants in only 4/26 papers; only 1/26 that blinded providers; 11/26 reporting on co-interventions; and 14/26 reporting on compliance.

Risk of detection bias was low with: blinding of outcome assessment in 20/26 papers; and timing of outcome assessments 21/26. With respect to attrition bias, incomplete outcome data and drop outs were reported in 20/26 studies and these were deemed to be at acceptable levels (< 20%). Intention to treat analysis was reported in 16/26 and unclear in 7/26 studies. Reporting bias (selective reporting) was unclear in 19/26 papers, with only 5/26 with published protocols or trial registration that could be sourced for comparison with the published results. Fifteen of 26 papers declared no conflicts of interest (other bias), however, in all the other studies possible conflicts and funding sources were not declared or it was unclear whether they could be a source of bias.

## Discussion

This systematic review aimed to explore the possible effect/s of manual therapy on various measures of balance and stability in people with musculoskeletal pain. Improvement in clinical balance measures were reported in studies comparing manual therapy in the short-term, but not at long-term follow-up. Likewise, objective balance measures showed improvements with interventions consisting of manual therapies in 5/8 studies. The most common presenting complaints included lower limb OA and LBP. Risk of bias was low across all reported criteria except for practitioner and participant blinding. It is noted that blinding of participants and providers is difficult and often not possible for providers of manual therapies.

Short-term follow-up on gait speed showed significant improvements associated with manual therapy compared to other interventions. Clinically meaningful change to gait speed has been previously calculated as a change larger than 0.05 m/s (indicating small change) and 0.1 m/s (indicating substantial change) [44]. This meta-analysis found that short-term interventions consisting of manual therapy, compared to other interventions, had a mean improvement of 0.09 m/s (95%CI 0.04–0.13). This indicates a relatively substantial improvement in gait speed, compared to the other interventions. However, these results should be interpreted with caution as there was a high level of statistical heterogeneity (i^2^ = 54%). Likewise, in short-term follow-up, manual therapy compared to other interventions showed a statistically significant improvement in TUG (mean improvement of − 0.53 s (95%CI -0.99—0.07). However, this improvement is significantly lower than the minimum clinically important difference of 3.4 s [45]. Furthermore, the study by Cortés Godoy and colleagues [26] (not included in the meta-analysis) did not find any significant difference between combined manual therapy and exercise and exercise alone on the get up and go test (a physical performance test similar to TUG) in short-term follow-up. Again, the heterogeneity in this meta-analysis was high (i^2^ = 52%). Therefore, we stress that these results are preliminary and should be interpreted with caution.

Falls are much more prevalent in older people with pain than in those without pain [4]. Recent epidemiological data out of Europe shows that chronic musculoskeletal pain is very frequent in older adults [1]. Therefore, any intervention that reduces pain intensity should be accompanied by a reduction in the rate of falls in older people. Further theoretical neuro-physiological associations between manual therapy and improved stability may be considered hypothetical as there is limited evidence on the mechanisms underlying the role of manual therapy on postural stability.

This systematic review had several strengths. These included capturing a broad range of studies measuring stability outcomes, across a range of musculoskeletal conditions, and types of manual therapies. This broad approach outlined clinically relevant evidence that may help to inform future studies of manual therapies with a suggestion to include clinical outcome measures beyond pain that also capture stability. Furthermore, this study used the Cochrane protocol to maintain scientific rigor.

This study also suffers from several weaknesses. We included studies based on diverse outcome measures which had high levels of heterogeneity across study design and data analysis, particularly co-interventions and comparison interventions. The inclusion of heterogeneous outcome measures meant that it was only possible to compare the studies in a narrative synthesis. Furthermore, this systematic review was not able to capture all potential modifiers or factors that may be associated with stability such as specific classifications of musculoskeletal conditions (eg OA, myofascial pain syndromes, biomechanical joint dysfunction), affected bodily regions (extremities and spinal), chronicity of these conditions (acute, subacute or chronic), participants’ response to therapies in isolation and in combination, and responses to therapies in the short, medium and long-term.

In analysing all the studies included in this review, we did not find a clear association between significant pain reduction and balance. Part of the explanation for this lack of association may be the heterogeneity of the conditions being investigated, particularly the site of pain. It is possible that pain reduction is driving some of the improvements in the clinical testing of stability, in particular gait speed as well as in objective static and dynamic balance testing.

Further prospective studies are recommended to explore if manual therapy should be provided alone or as part of other interventions, and at what point manual therapy should be transitioned to more active interventions in the longer term. For example, if a patient is unable to exercise because of a painful musculoskeletal condition, is there a possible benefit in initially managing the condition with passive manual therapies to decrease their pain so that they might transit to appropriate exercise therapy? This is particularly important for studies in older populations where pain is a barrier to undertaking an exercise plan [46]. The effects of treatment dose and duration also need to be explored, as higher doses of manual therapy or more extended treatment regimens may have different relationships to pain reduction and possible improvement in postural or dynamic stability. Finally, this review recommends that studies of interventions for musculoskeletal pain should include outcome measures of stability, particularly in studies including older people.

Given the substantial burden of illness caused by falls in our ageing population a better understanding of how to modify risk factors including gait speed via appropriate management strategies requires further exploration.

## Data Availability

The datasets used and/or analysed during the current study are available from the corresponding author on reasonable request.
